# SCAP Mediated GDF15-Induced Invasion and EMT of Esophageal Cancer

**DOI:** 10.3389/fonc.2020.564785

**Published:** 2020-10-06

**Authors:** Gang Dong, Xiaoquan Huang, Siyu Jiang, Liyuan Ni, Lili Ma, Chouwen Zhu, Shiyao Chen

**Affiliations:** ^1^Department of Gastroenterology and Hepatology, Zhongshan Hospital, Fudan University, Shanghai, China; ^2^Endoscopy Center and Endoscopy Research Institute, Zhongshan Hospital, Fudan University, Shanghai, China; ^3^Center of Evidence-Based Medicine, Fudan University, Shanghai, China

**Keywords:** esophageal cancer, metastasis, GDF15, SCAP, miR-1324

## Abstract

**Background:** GDF15 is a potential biomarker for patients with esophageal cancer (EC). However, the mechanistic role of GDF15 in the invasion and metastasis of EC remains poorly understood.

**Methods:** We determined the expression and function of GDF15 in esophageal cancer cells (ESCCs) and in patient tissue samples using western blotting, migration, and invasion assays, immunohistochemistry, Co-IP assays, and quantitative real-time-PCR. In addition, a pulmonary metastatic nude mouse model was used to determine the function of GDF15. We then supplemented our experimental results with database analysis to validate our findings.

**Results:** GDF15 was upregulated in EC, and was associated with poor differentiation, high metastasis rates, and worse prognosis. GDF15 knock-down reduced the migration and invasion of ESCCs. Co-IP assays demonstrated its association with SCAP, while GDF15 knock-down decreased SCAP levels. SCAP overexpression reversed the migration, invasion and EMT in GDF15-siRNA ESCCs. However, after incubation with β-cyclodextrin (β-CD), the ability of migration and invasion was weakened, EMT was reversed again. Migration, invasion, and EMT were enhanced in GDF15-siRNA ESCCs cultured in the presence of cholesterol and were similar to GDF15-siRNA ESCCs overexpressing SCAP. *In vivo*, knockdown of GDF15 inhibited lung metastasis of ESCCs and was reversed by SCAP overexpression or high cholesterol diet. Increased lung metastasis after SCAP overexpression was partially suppressed by intraperitoneal injection of β-CD. In addition, we determined that GDF15 was a direct target of miR-1324, miR-1324 was down-regulated in EC tissues. MiR-1324 upregulation resulted in decreased GDF15 expression and metastasis in ESCCs.

**Conclusions:** We demonstrated that SCAP mediated GDF15-induced the invasion and metastasis of EC by regulating cholesterol metabolism. In addition, GDF15 was shown to be a direct target of miR-1324.

## Introduction

EC is a common malignant tumor with a worldwide prevalence (1). An estimated 246,000 new cases were diagnosed in China in 2015, with approximately 188,000 dying from the disease (2). The 5-year overall survival rate is below 20% despite improvements in medical facilities and treatment methods. The poor prognosis of EC is largely due to its high metastatic rates (3). Hence, it is important to identify the molecular mechanisms underlying metastasis of EC to aid in the development of new treatment strategies for EC patients.

Growth differentiation factor 15 (GDF15), is a divergent member of the TGF-β superfamily. GDF15 is also known as macrophage inhibitory cytokine-1(MIC-1) or non-steroidal anti-inflammatory drug-activated gene-1 (NAG-1). Inflammation, obesity, cardiovascular diseases, and tumors have been shown to induce abnormal expression of GDF15 (4, 5). Previous studies have shown that GDF15 expression is elevated in certain human tumors, and dysregulation of GDF15 has been correlated with tumor progression and poor clinical outcomes (6–9). GDF15 has served as a potential biomarker for patients with EC (10). However, the biological function of GDF15 in EC remains poorly understood. Previous reports regarding the role of GDF15 in the invasion and metastasis of EC are rare, with the underlying mechanism yet to be deciphered.

TGFβ1 has been reported to activate the sterol regulatory element-binding protein 2 (SREBP2) in kidney mesangial cells, and SREBF2 activation was dependent on sterol regulatory element-binding protein cleavage-activating protein (SCAP) (11, 12), inhibition of SCAP or SREBPs significantly suppressed tumor growth in various cancer (13). As a divergent member of the TGF-β superfamily, GDF15 may also promote the progression of EC through SCAP/SREBPs pathway. SREBF2 is mainly responsible for cholesterol synthesis and uptake, cholesterol could promote colorectal cancer progression, and inhibit cell apoptosis (14). Cholesterol levels are higher in EC tissues compared to corresponding tumor-adjacent tissues (15). To the best of our knowledge, there have been only a few reports regarding the role of SCAP or cholesterol in the metastasis of EC. After analyzing protein interaction databases, we found an interaction between GDF15 and SCAP. Therefore, the present study focused on the importance of this interaction to determine the underlying mechanism of GDF15 in the metastasis of EC

In addition to regulating SCAP/SREBF2 pathway, GDF15 itself may also be a target of some miRNAs, which involved in the post-transcriptional regulation of gene expression by the binding of miRNAs to the 3′-untranslated region (3′-UTR) of target mRNAs (16). Aberrant expression of specific miRNAs have been implicated in EC development and progression, i.e., miR-450a-5p has been shown to inhibit autophagy and enhance radio-sensitivity in EC (17), while miR-338-5p has been shown to reverse chemoresistance and inhibit invasion of EC (18). The GDF15 mRNA transcript has a putative target sequence for miR-1324 in the 3′ UTR and has been predicted to be a direct target of miR-1324 using Targetscan. MiR-1324 has been previously identified as a tumor suppressor in hepatocellular carcinoma growth and metastasis (19). Whether miR-1324 may also be dysregulated and function as a GDF15 regulator in EC is yet to be deciphered.

Taken altogether, this study investigated the clinical significance and biological function of GDF15 in EC. We demonstrated that GDF15 downregulation inhibited tumor metastasis. Mechanistically, GDF15 regulated intracellular cholesterol levels by interacting with SCAP. The GDF15/SCAP/SREBF2 axis promoted migration and invasion of ESCCs and was targeted by miR-1324. The results of our study indicated the significance of the GDF15/SCAP/SREBF2 axis for EC metastasis and suggested that this pathway could be therapeutically targeted.

## Materials and Methods

### Antibodies and Reagents

The following antibodies were used for western blot analysis; anti-GDF15 antibody (cat.no. ab223539; Abcam), anti-snail antibody (cat.no. ab53519; Abcam), anti-slug antibody (cat.no. ab106077; Abcam), anti-β-actin antibody (cat.no. ab8224; Abcam), anti-E-cadherin antibody (cat.no. ab40772; Abcam), anti-Vimentin antibody (cat.no. ab92547; Abcam), anti-SCAP antibody (cat.no. ab125186; Abcam), anti-SREBF2 antibody (cat.no. ab30682; Abcam), and anti-HMGCR antibody (cat.no. ab174830; Abcam). Antibodies used for immunohistochemistry analysis were as follows; anti-GDF15 antibody (cat.no. ab223539; Abcam), anti-E-cadherin antibody (cat.no. ab40772; Abcam), anti-Vimentin antibody (cat.no. ab92547; Abcam), anti-SCAP antibody (cat.no. ab125186; Abcam), anti-SREBF2 antibody (cat.no. ab30682; Abcam), and anti-HMGCR antibody (cat.no. ab174830; Abcam). β-cyclodextrin (β-CD) and Cholesterol-Water Soluble powder was purchased from Sigma-Aldrich (St. Louis, MO). Recombinant human GDF15 (rhGDF15) was purchased from R&D Systems (Minneapolis, MN). Matrigel was purchased from Advanced BioMatrix (San Diego, CA). High-cholesterol diet was purchased from Trophic Animal Feed High-Tech Co., Ltd. (Nantong, China).

### Tumor Samples

This study was approved by the Ethics Committee of Zhongshan Hospital of Fudan University, Shanghai, China (B2016-100). EC tumor and matched para-cancer tissues were obtained from twenty patients undergoing treatment at the Zhongshan Hospital of Fudan University, from 2018 to 2019. Follow-up and clinical pathology data were collected from these twenty patients. All tissues were stored at −80°C until required.

### Cell Culture

ESCCs KYSE 150 and EC 9706 (Chinese Academy of Science cell bank, Shanghai, China) were cultured using Roswell Park Memorial Institute-1640 media (RPMI-1640; Invitrogen; Thermo Fisher Scientific, Inc., USA) supplemented with 10% fetal bovine serum and 1% penicillin-streptomycin. HET-1A (ATCC® CRL-2692™, ATCC) were cultured in Bronchial Epithelial Cell Basal Medium (BEBM) along with all the additives (cat no. CC-3170, Lonza/Clonetics Corporation). Cells were cultured in a humidified atmosphere of 5% CO2 at 37°C.

### Reverse Transcription-Quantitative Polymerase Chain Reaction (RT-qPCR)

Total cellular RNA was extracted using TRIzol (Invitrogen; Thermo Fisher Scientific, Inc.). The All-in-One™ miRNA qRT-PCR reagent kit (GeneCopoeia Inc. Maryland) was used to reverse-transcribe total cellular RNA into cDNA and then amplified using miRNA specific primers. Target gene expression was quantified using the 2^−ΔΔ*Cq*^ method and normalization to U6 levels.

### Western Blot Analysis

RIPA buffer containing PMSF (Beyotime Institute of Biotechnology) was used to lyse cells on ice. The BCA protein assay (Pierce; Thermo Fisher Scientific, Inc.) was then used to measure the protein concentration of the samples. SDS-PAGE was used to separate total protein (20 μg/well). Proteins were then transferred onto a PVDF membrane (EMD Millipore), blocked using 5% low-fat milk for 1 h, and then incubated with the relevant primary antibodies at 4°C overnight. The next day, membranes were incubated with secondary antibodies (Beyotime Institute of Biotechnology) for 1 h at 37°C. Protein bands were visualized using ECL (Pierce; Thermo Fisher Scientific, Inc.). Target protein levels were normalized to β-actin levels.

### Migration and Invasion Assays

Cellular migration and invasion were determined using Transwell Chambers (Corning Inc., USA). Briefly, 5 × 10^4^ cells were added to the upper chamber (Matrigel (Corning Inc., USA), (diluted 1:9) pre-coated for invasion) with 200 μl of serum-free media. The bottom chamber contained 600 ul of complete media. After 24 h, cells on the upper surface of the membrane were removed, and cells that had migrated to the lower membrane were fixed and stained. Cell numbers were analyzed using a microscope (Leica Microsystems, Germany).

### Transfection

siRNA against human GDF15 (hU6-MCS-CBh-gcGFP-IRES-puromycin) and the appropriate scramble control siRNA were purchased from Genechem Co. Ltd. (Shanghai, China). ESCC cells at 30–50% confluence were transfected with lentivirus vector in the presence of 5 μg/mL of polybrene for 24 h. Lentivirus-mediated overexpression of SCAP (Ubi-MCS-3FLAG-CBh-gcGFP-IRES-puromycin) and the appropriate negative control was purchased from Genechem Co. Ltd. (Shanghai, China). ESCC cells at 30–50% confluence were infected with lentivirus in the presence of 5 μg/mL of polybrene for 24 h. MiR-1324 mimics (5′CCAGACAGAAUUCUAUGCACUUUC3′, 5′AAGUGCAUAGAAUUCUGUCUGGUU3′) and the negative control were obtained from (GeneCopoeia Inc. Maryland). ESCC cells at 50–80% confluence were transfected with the mimics in the presence Lipofectamine 2000 (Invitrogen, Carlsbad, CA, USA) according to the manufacturer's instructions for 48 h. Lentivirus-mediated overexpression of miR-1324 (hU6-MCS-Ubiquitin-EGFP-IRES-puromycin) and negative control was obtained from Genechem Co. Ltd. (Shanghai, China). ESCC cells at 30–50% confluence were infected with lentivirus in the presence of 5 μg/mL of polybrene for 24 h.

### Immunohistochemistry

Lung tissues from nude mice and clinical samples were fixed at room temperature with 4% paraformaldehyde for ~ 10 h before paraffin embedding. Tissues were then sectioned into 4-μm thick slices. Deparaffinization and rehydration were performed using xylene and a graded series of ethanol, respectively. Hematoxylin and Eosin (H&E) (Beyotime Institute of Biotechnology) was used to stain lung tissues. Clinical samples were incubated with specific primary antibodies against GDF15 (cat.no. ab223539; Abcam), E-cadherin (cat.no. ab40772; Abcam), Vimentin (cat.no. ab92547; Abcam), SCAP (cat.no. ab125186; Abcam), SREBF2 (cat.no. ab30682; Abcam), or HMGCR (cat.no. ab174830; Abcam) at 4°C overnight followed by incubation with the appropriate secondary antibody (cat. no. A0208, Beyotime Institute of Biotechnology) for 30 min at room temperature. DAB was used as the chromogen. Images were captured using a microscope (Leica Microsystems, Germany).

### Co-immunoprecipitation (CoIP) Assays

Total proteins were extracted using the Thermo Scientific IP Lysis Buffer (cat. no. 87787) containing PMSF. Cell lysates were incubated with antibodies against GDF15 (cat.no. ab223539; Abcam) or SCAP (cat.no. ab125186; Abcam) at 4°C on a shaker overnight, followed by incubation with 50 μl Protein A/G PLUS-Agarose beads (cat. no. sc-2003; Santa Cruz Biotechnology, Inc.) for an additional 10 h. Beads were then washed 4 times with 1 mL Lysis Buffer before denaturation using 2 × SDS sample buffer at 100°C for 20 min for subsequent western blot assays.

### Dual-Luciferase Reporter Assay

Target genes of miR-1324 were identified using Targetscan. Wild-type and mutant 3′UTR sequence of GDF15 were synthesized and inserted into the pGL3 promoter vector (WT:PGL3-CMV-LUC-H_GDF15; MT:PGL3-CMV-LUC-H_GDF15) (Genomeditech Co. Ltd.). Cells were co-transfected with the 3′-UTR WT or MT plasmid with miR-1324 or negative control using Lipofectamine 2000 (Invitrogen, Thermo Fisher Scientific, Inc.). Forty eight hours post-transfection, relative luciferase activity was measured using the Dual-Luciferase® Reporter Assay System (Promega, Madison, WI, USA) according to the manufacturer's instructions. Firefly luciferase activity was normalized to Renilla luciferase activity.

### RNA Fluorescence *in situ* Hybridization (FISH)

miR-1324 and GDF15 expression in EC specimens were determined using FISH. Specific probes were purchased from RiboBio (Guangzhou, China). After tissues were deparaffinized and dehydrated as described above, sections were hybridized with the specific probes in hybridization buffer overnight at 37°C in a dark moist chamber. The next day, slides were washed and counterstained with DAPI. Images were captured using a fluorescence microscope (Leica Microsystems, Germany).

### Animal Experiments

All animal experiments were approved by the ethics committee of Zhongshan Hospital of Fudan University, Shanghai, China. This study was performed according to the guidelines of the Shanghai Medical Experimental Animal Care Commission. Tumor implantation was performed using KYSE150 cells, which included cells transfected with NC-siRNA + NC-OE, GDF15-siRNA + NC-OE, and GDF15-siRNA + SCAP-OE. 1 × 10^6^ cells were injected into the tail veins of BALB/c nude mice (18–20 g, 4–6 weeks). Two weeks later, mice injected with GDF15-siRNA + NC-OE and GDF15-siRNA + SCAP-OE were randomly divided into two groups, resulting in five groups of *n* = 5 for each group. Mice in the GDF15-siRNA + NC-OE group were fed a modified cholesterol-rich diet containing 2% cholesterol (cat.no. TP 06106; Trophic Animal Feed High-tech Co., Ltd. Nantong, China), while the remaining groups were fed a control diet for 8 weeks. One group of GDF15-siRNA + SCAP-OE mice were intraperitoneally injected with β-CD, while the other groups were administered a vehicle control twice a week. Two months later, mice were euthanized, and the lungs harvested for H&E staining.

### Statistical Analysis

Data analysis was performed using the SPSS 24.0 software (SPSS, Inc.). Mean ± standard deviation was used to represent all values. One-way analysis of variance or student's unpaired *t-*test was used to compare three or two groups, respectively. *P* < 0.05 was considered statistically significant.

## Results

### GDF15 Expression Levels in EC and Adjacent Tissues

**Figure 1:**
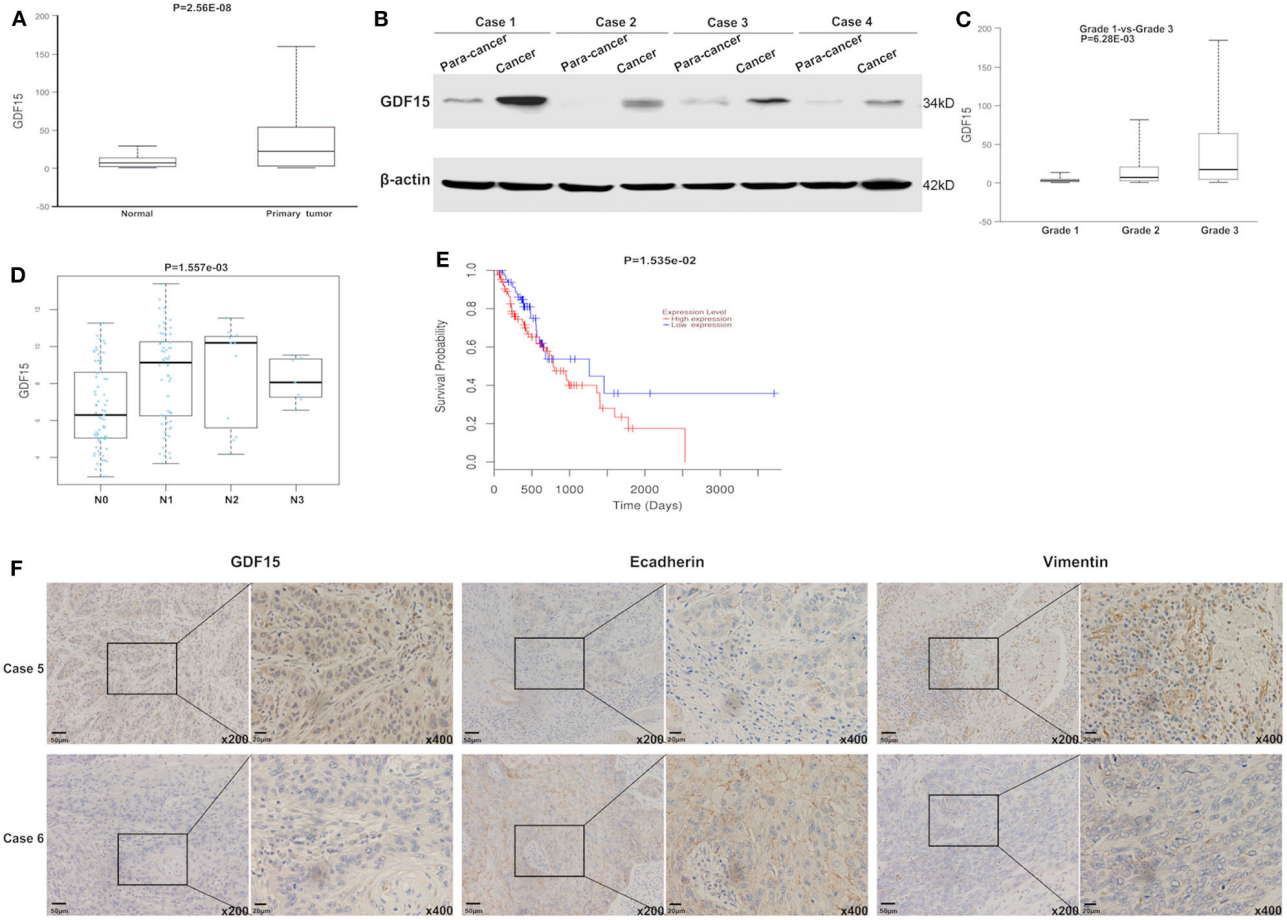
GDF15 expression levels in EC and adjacent tissue. **(A)** GDF15 expression levels were analyzed using the EC dataset in TCGA. GDF15 expression levels in EC and adjacent tissues determined by western blotting **(B)**. **(C–E)** Correlation between GDF15 expression levels and tumor grade, lymphatic metastasis, and overall survival in EC analyzed using TCGA. **(F)** Expression levels of GDF15, E-cadherin, and vimentin in EC and adjacent tissues determined by immunohistochemistry.

Using the gene expression TCGA database, we observed that GDF15 expression was higher in EC compared to adjacent normal tissue (Figure 1A). This was consistent with our western blot results (Figure 1B). High GDF15 expression levels predicted poor differentiation, higher metastasis rates and worse prognosis in patients with EC (Figures 1C–E). In addition, GDF15 expression levels were negatively correlated with E-cadherin and positively correlated with vimentin (Figure 1F). Hence, we hypothesized that GDF15 may be involved in invasion and metastasis of EC.

### GDF15 Induced Migration and Invasion of ESCCs

**Figure 2:**
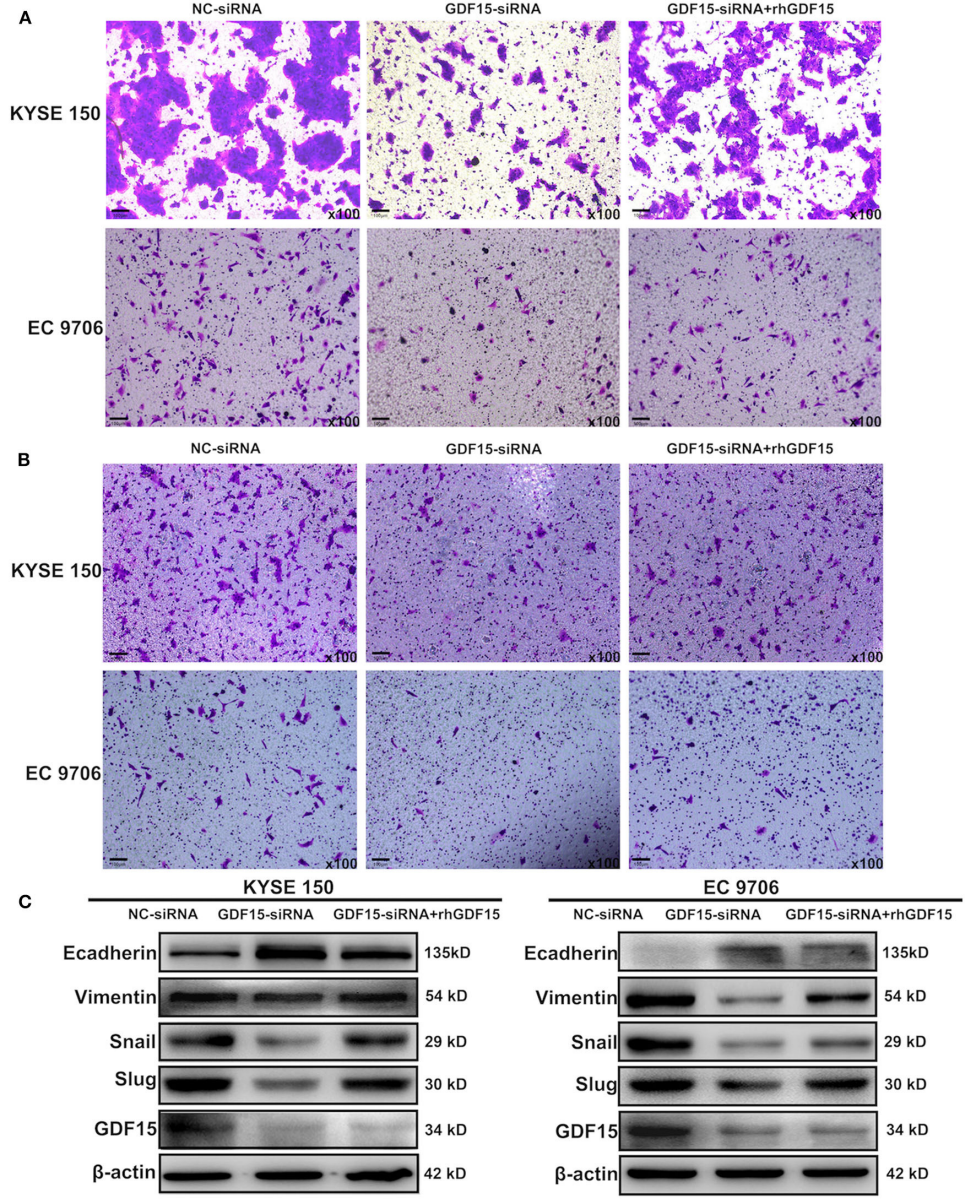
Role of GDF15 in invasion and EMT of ESCCs (NC-siRNA, GDF15-siRNA, GDF15-siRNA + rhGDF15). **(A,B)** Effect of GDF15 on migration and invasion determined using transwell chamber assays. **(C)** Expression levels of EMT related proteins E-cadherin, vimentin, snail, and slug determined by western blot.

Transwell chambers were used to determine the migration and invasion ability of ESCCs. As shown in Figures 2A,B, GDF15-siRNA ESCCs had reduced migration and invasion ability but was restored after incubation with rhGDF15 (100 ng/ml) for three culture generations. Target genes associated with EMT were then analyzed. As shown in Figure 2C, vimentin, snail, and slug levels were decreased and E-cadherin levels were increased in GDF15-siRNA ESCCs, suggesting reduced EMT. After incubation with rhGDF15, their expression levels were restored. These results suggest that GDF15 promotes migration and invasion of ESCCs.

### SCAP Mediated GDF15-Induced Migration and Invasion of ESCCs

**Figure 3:**
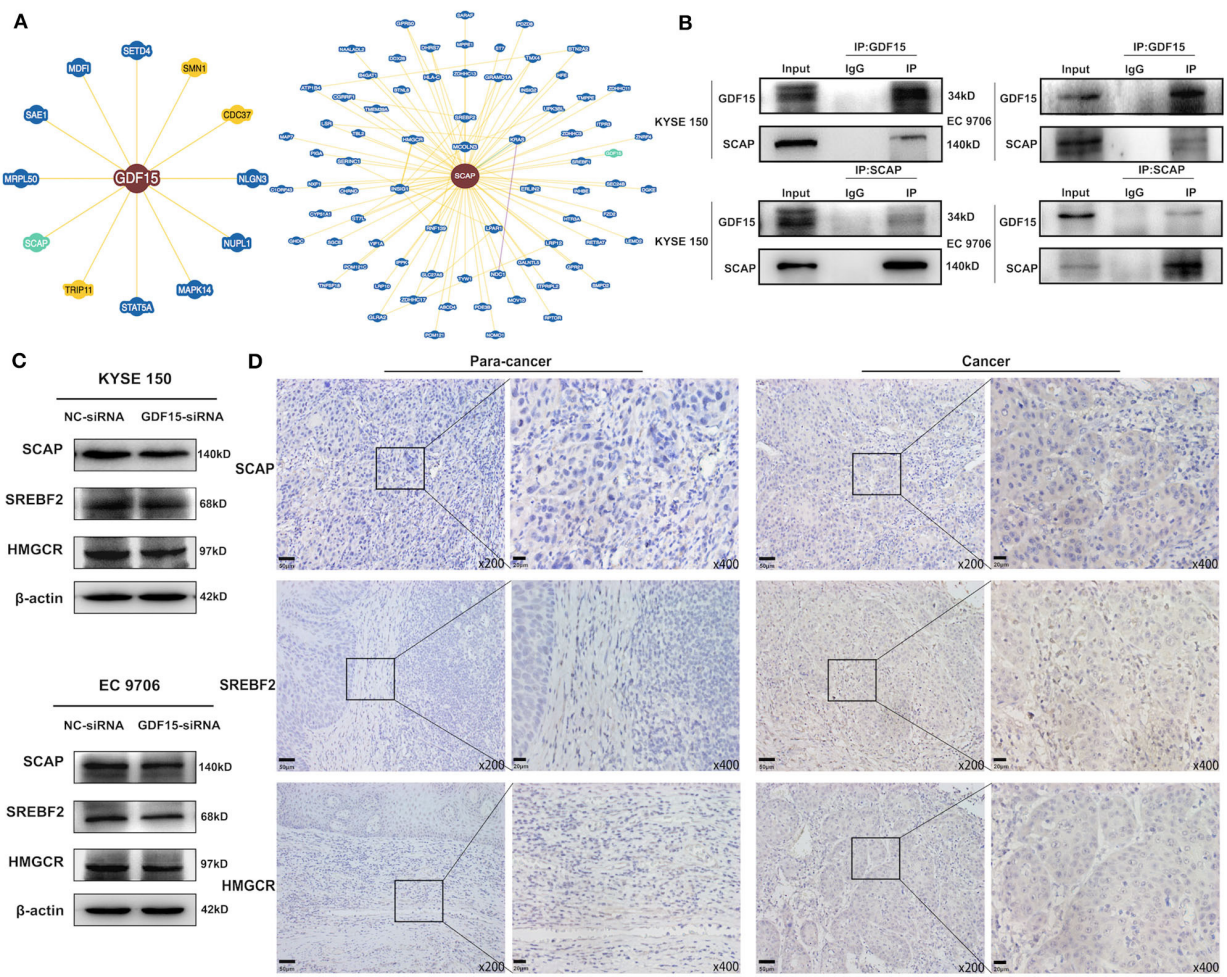
Identification of GDF15–SCAP protein interactions. **(A,B)** Analysis of protein interactions between GDF15 and SCAP using the BioGRID database. CoIP assays were used to verify these interactions. **(C)** SCAP, SREBF2, and HMGCR expression levels in GDF15-siRNA ESCCs determined by western blotting. **(D)** SCAP, SREBF2, and HMGCR expression levels in EC and adjacent tissues determined by immunohistochemistry.

**Figure 4:**
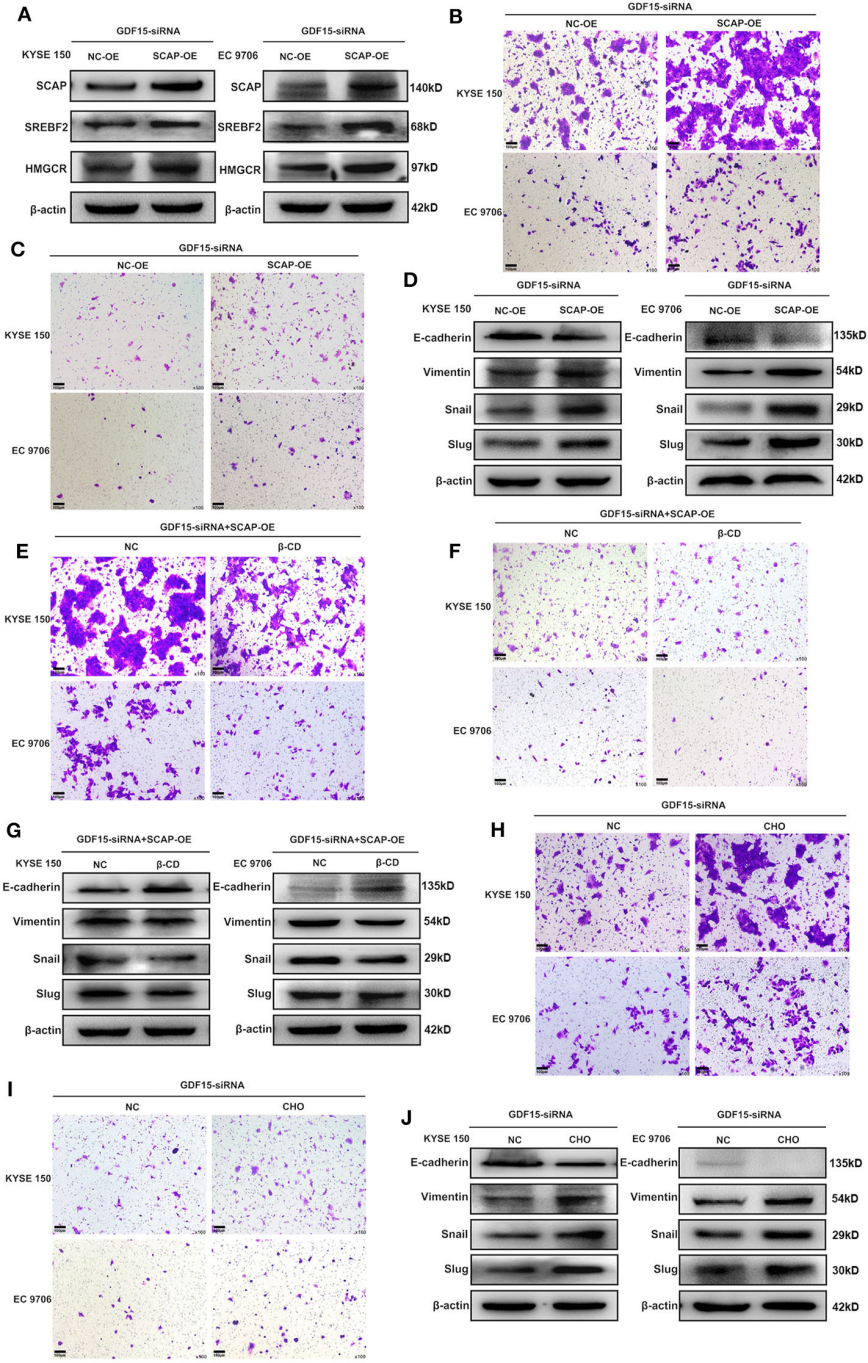
SCAP mediated GDF15-induced migration and invasion of ESCCs via cholesterol metabolism. **(A)** Expression levels of SREBF2 and HMGCR in SCAP-overexpressing GDF15-siRNA ESCCs determined by western blotting. **(B–G)** SCAP overexpression in GDF15-siRNA ESCCs induced cell migration, invasion, and EMT, which was reduced by β-CD treatment (2.5 mM). **(H–J)** Pretreatment with cholesterol (5 ug/ml) induced cell migration, invasion, and EMT of GDF15-siRNA ESCCs.

It has been previously reported that GDF15 could promote colon cancer invasion and metastasis by activating c-Fos (20). We investigated whether GDF15 played an additional role in EMT and metastasis of EC. As shown in Figure 3A, an interaction between GDF15 and SCAP was observed using the protein interaction database. This interaction was confirmed using CoIP assays (Figure 3B). Interestingly, the expression of SCAP and its downstream target genes, i.e., SREBF2 and HMGCR were reduced in siRNA-GDF15 ESCCs (Figure 3C). In addition, SCAP, SREBF2, and HMGCR expression levels in EC were higher compared to adjacent normal tissues (Figure 3D). We observed that SCAP overexpression could up-regulate SREBF2 and HMGCR expression levels (Figure 4A), and reverse the migration and invasion ability of GDF15-siRNA ESCCs (Figures 4B,C), increase the expression of vimentin, snail, and slug, and decrease E-cadherin expression (Figure 4D). One of the main functions of SCAP is to regulate intracellular cholesterol levels (21). SCAP overexpression has been reported to promote the accumulation of intracellular cholesterol in THP-1 macrophages (22). To demonstrate the role of SCAP in promoting migration, invasion, and EMT of GDF15-siRNA ESCCs, β-CD, a reagent used to deplete intracellular cholesterol levels, was added to GDF15-siRNA ESCCs overexpressing SCAP. As shown in Figures 4E–G, after β-CD incubation, migration and invasion ability were reduced, and EMT was reversed. In addition, invasion and EMT were enhanced after GDF15-siRNA ESCCs were incubated with cholesterol (Figures 4H–J), and were similar to GDF15-siRNA ESCCs overexpressing SCAP. These results suggest that SCAP mediated GDF15-induced migration and invasion of ESCCs was through cholesterol metabolism.

### GDF15/SCAP/SREBF2 Axis Was Targeted by miR-1324

**Figure 5:**
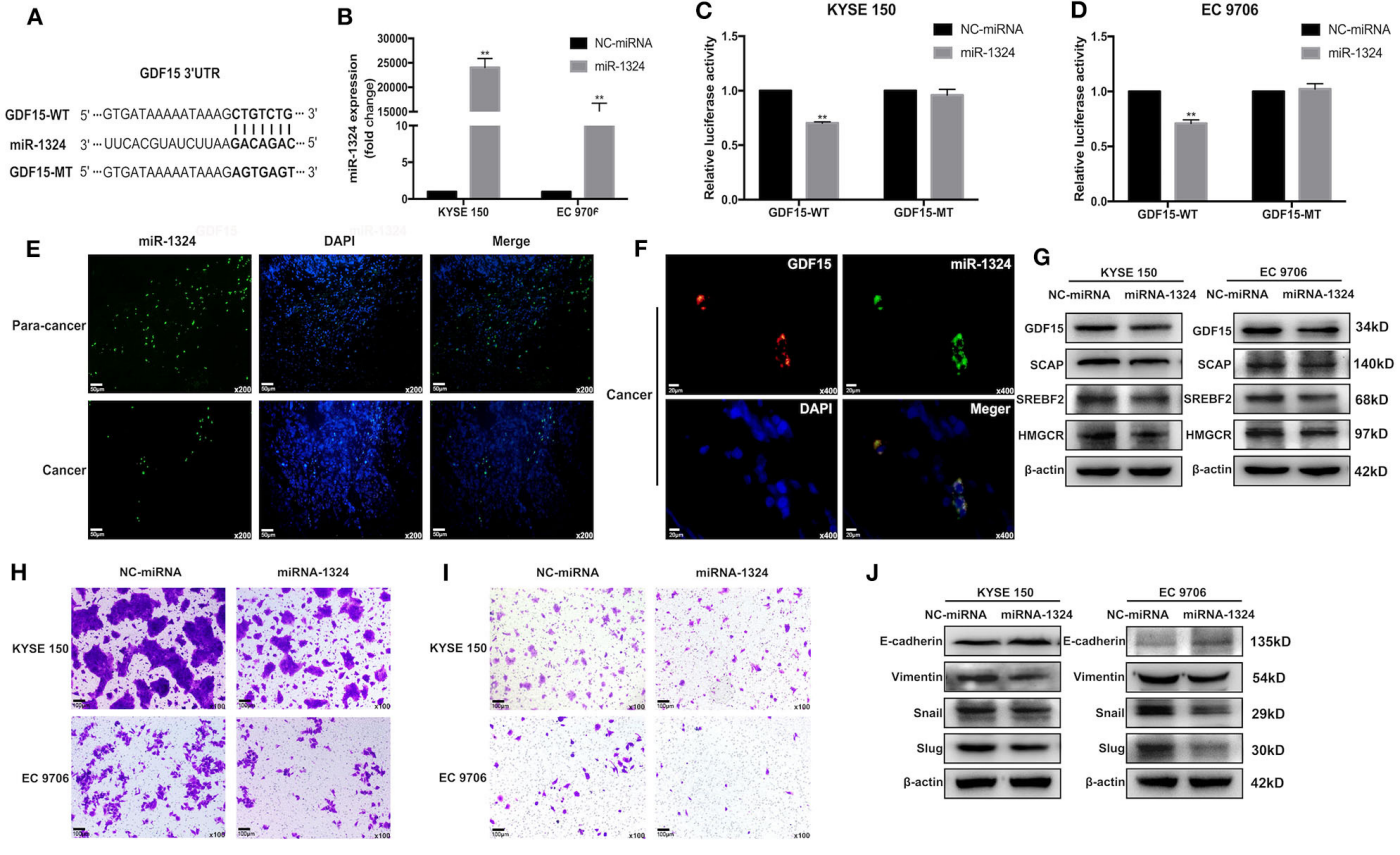
GDF15 is a direct target of miR-1324. **(A)** A schematic of the target sites (wild and mutant) of miR-1324 in the 3′ UTR of the GDF15 mRNA transcript. **(B)** After the transfection of miR-1324 mimics, miR-1324 expression in ESCCs was determined by RT-PCR. **(C,D)** Dual-Luciferase reporter assays performed in ESCCs co-transfected with 3′-UTR WT or MT plasmid with miR-1324 or negative control using Lipofectamine 2000. Relative luciferase activity was measured using the Dual-Luciferase® Reporter Assay System. **(E)** Expression of miR-1324 (green) in EC and adjacent non-tumor tissues were determined by FISH, nuclei were stained with DAPI (blue). **(F)** Subcellular co-localization analysis of the interaction between GDF15 (red) and miR-1324 (green) in EC tissues, nuclei were stained with DAPI (blue). **(G)** Expression levels of SCAP, SREBF2, and HMGCR determined by western blotting. **(H–J)** Migration and invasion of ESCCs after transfection with miR-1324 mimics were determined using transwell chamber assays. Expression levels of EMT-associated proteins E-cadherin, vimentin, snail, and slug were determined by western blotting. **P < 0.01.

Accumulating evidence has demonstrated that miRNAs play an important role in the progression of human cancers (23). GDF15 mRNA transcript has a putative target sequence for miR-1324 in the 3′ UTR and was predicted to be a direct target of miR-1324 by Targetscan (Figure 5A). We validated this finding using luciferase reporter assays (Figures 5C,D). In addition, subcellular co-localization analyses demonstrated a cytoplasmic interaction between GDF15 and miR-1324 in EC tissues (Figure 5F). The function and molecular mechanism of miRNA-1324 in EC were then investigated. As shown in Figure 5E, the expression of miR-1324 in adjacent non-tumor tissues was higher compared to EC tissues. After transfection with miR-1324 mimics, miR-1324 expression levels increased in ESCCs (Figure 5B), while there was a decrease in GDF15, SCAP, SREBF2, and HMGCR expression levels and reduced migration, invasion, and EMT (Figures 5G–J). These results suggest that miR-1324 could reduce the invasion and metastatic ability of ESCCs by targeting GDF15.

### GDF15 Knockdown Reduced Lung Metastasis *in vivo*

**Figure 6:**
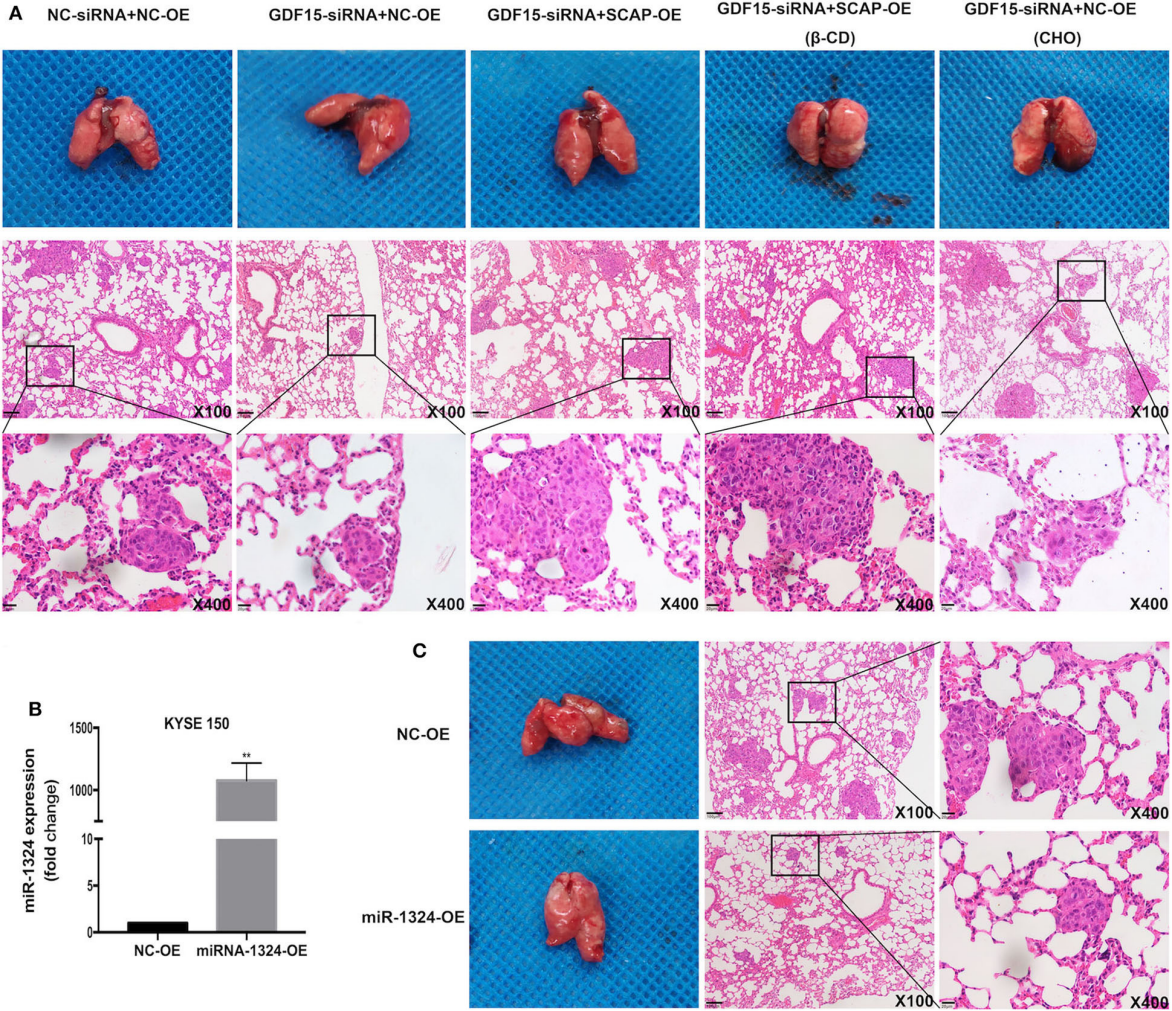
SCAP mediated GDF15-induced lung metastasis of KYSE 150 via cholesterol metabolism. **(A)** Reduced lung metastasis was observed in mice transplanted with GDF15 knockdown KYSE 150 cells, which was reversed by SCAP overexpression or high cholesterol diet. Lung metastasis in transplanted SCAP overexpressing cells was partially reduced by intraperitoneal injection of β-CD (0.5 g/kg). **(B)** MiR-1324 expression in KYSE 150 cells transfected with lentivirus overexpressing miR-1324 determined by RT-PCR. **(C)** Mice injected with KYSE 150 cells transfected with lentivirus overexpressing miR-1324 had reduced lung metastasis. **P < 0.01.

Finally, we evaluated the effect of GDF15 *in vivo* by subcutaneously injecting KYSE 150 cells in nude mice. Knockdown of GDF15 expression inhibited KYSE 150 cells lung metastasis and was reversed by SCAP overexpression or high cholesterol diet. The effect of SCAP overexpression on lung metastasis was partially offset by the intraperitoneal injection of β-CD (Figure 6A). Mice injected with KYSE 150 cells transfected with lentivirus overexpressing miR-1324 had reduced lung metastasis (Figures 6B,C). These results indicate that GDF15 was involved in the lung metastasis of EC through SCAP-regulated cholesterol and was suppressed by miR-1324 *in vivo*.

## Discussion

EC is a kind of human malignancy that is prevalent worldwide and endangers human life and health (1). It is characterized by an insidious onset, low survival rate, high morbidity, and mortality. The 5-year overall survival rate is below 20%, with poor prognosis closely correlated with its high metastatic rate (3). Hence, it is important to identify the underlying mechanisms of metastatic EC. A previous study indicated that GDF15 could serve as a potential biomarker for EC (10). GDF15 not only promoted bone metastasis of prostate cancer but also induced colon cancer formation in senescence-associated tissue microenvironment (6, 24). However, the role of GDF15 in the invasion and metastasis of EC remains poorly understood. Both TGFβ1 and GDF15 belongs to TGF-β superfamily, TGFβ1 was reported to activate SREBP2 in kidney mesangial cells, SREBF2 activation was dependent on SCAP (11, 12), SCAP or SREBPs promoted tumor progression in various cancer (13). Therefore, we speculated that GDF15 may also regulate the invasion and metastasis of EC through SCAP/SREBF2 axis, which involved cholesterol synthesis and uptake. Besides regulating SCAP/SREBF2 pathway, GDF15 may be also targeted by certain miRNAs, until now, upstream miRNAs targeting GDF15 have not been clarified. Hence, the present study investigated that GDF15 regulated intracellular cholesterol levels by interacting with SCAP/SREBF2. The GDF15/SCAP/SREBF2 axis promoted migration and invasion of ESCCs and was targeted by miR-1324.

As a divergent member of the TGF-β superfamily, GDF15 is considered to be a promoter for the development and progression of some cancers. Additionally, it plays a role in apoptosis resistance, invasion, and metastasis of tumors (25–27). In the present study, we observed that GDF15 expression levels in EC were higher compared to adjacent normal tissues (Figures 1A,B). In addition, high expression levels predicted poor differentiation, higher aggressiveness, and worse prognosis in EC patients (Figures 1C–E). EMT is a process characterized by loss of cell-cell adhesion and increase migration and invasion ability. The trans-differentiation from quiescent epithelial cells into motile mesenchymal cells is essential for tumor progression (28, 29). We observed that GDF15 expression levels were negatively correlated with adhesion molecule E-cadherin levels and positively correlated with mesenchymal marker vimentin (Figure 1F). To further understand the biological role of GDF15 in the invasion and metastasis of esophageal carcinoma, ESCCs were transfected with siRNA against GDF15. As shown in Figure 2, migration and invasion ability were reduced and EMT was inhibited in GDF15-siRNA ESCCs. This was reversed when cells were incubated with rhGDF15. These results were further confirmed using *in vivo* mouse models (Figure 6A).

Next, we investigated the mechanism of GDF15 to promote invasion and metastasis of EC. We observed an interaction between GDF15 and SCAP (Figures 3A,B). TGFβ1 has been reported to activate SREBF2 in kidney mesangial cells, and SREBF2 activation was dependent on SCAP (11, 12). In this study, knock-down of GDF15 decreased expression of SCAP, SREBF2, and HMGCR in siRNA-GDF15 ESCCs (Figure 3C), while SCAP overexpression could increase SREBF2 and HMGCR expression levels (Figure 4A) and reverse the migration, invasion, and EMT of GDF15-siRNA ESCCs (Figures 4B–D). As a sterol sensor protein, SCAP forms a complex with SREBP2 to regulate its activation. This in-turn mediates *de novo* synthesis and uptake of cholesterol (21, 30). To further understand the role of SCAP in enabling invasion and migration of GDF15-siRNA ESCCs, β-CD was used to deplete intracellular cholesterol levels in GDF15-siRNA ESCCs overexpressing SCAP. β-CD incubation reduced the effect induced by SCAP overexpression in GDF15-siRNA ESCCs (Figures 4E–G). Cholesterol addition restored migration, invasion and EMT in GDF15-siRNA ESCCs (Figures 4H–J). *In vivo* animal experiments validated our findings (Figure 6A).

GDF15 was not only involved in the control of intracellular cholesterol level through SCAP/SREBF2 pathway to promote EC progression, but also negatively regulated by upstream miRNAs. Aberrant miRNA expression has been reported to play an important role in EC. MiR-203a has been shown to inactivate the PI3K/AKT/mTOR pathway and inhibit EC development (31), while microRNA-10b induces cisplatin resistance by targeting PPARγ in EC (32). However, the function and molecular mechanism of miRNA-1324 in EC progression are yet to be deciphered. The GDF15 mRNA transcript has a putative target sequence for miR-1324 in the 3′ UTR and was predicted to be a direct target of miR-1324 using Targetscan (Figure 5A). We validated these findings using luciferase reporter assays (Figures 5C,D). miR-1324 expression levels in adjacent non-tumor tissues were higher compared to EC tissues. After transfection with miR-1324 mimics, miR-1324 expression levels increased in ESCCs (Figure 5B), expression levels of GDF15, SCAP, SREBF2, and HMGCR decreased, migration and invasion were inhibited, and EMT reversed (Figures 5G–J). *In vivo*, mice injected with ESCCs transfected with lentivirus overexpressing miR-1324 had reduced lung metastasis (Figures 6B,C). miR-1324 was identified as a tumor suppressor in hepatocellular carcinoma growth and metastasis (19). Our results suggest that miR-1324 could reduce the invasion and metastasis of ESCCs by targeting GDF15.

**Figure 7:**
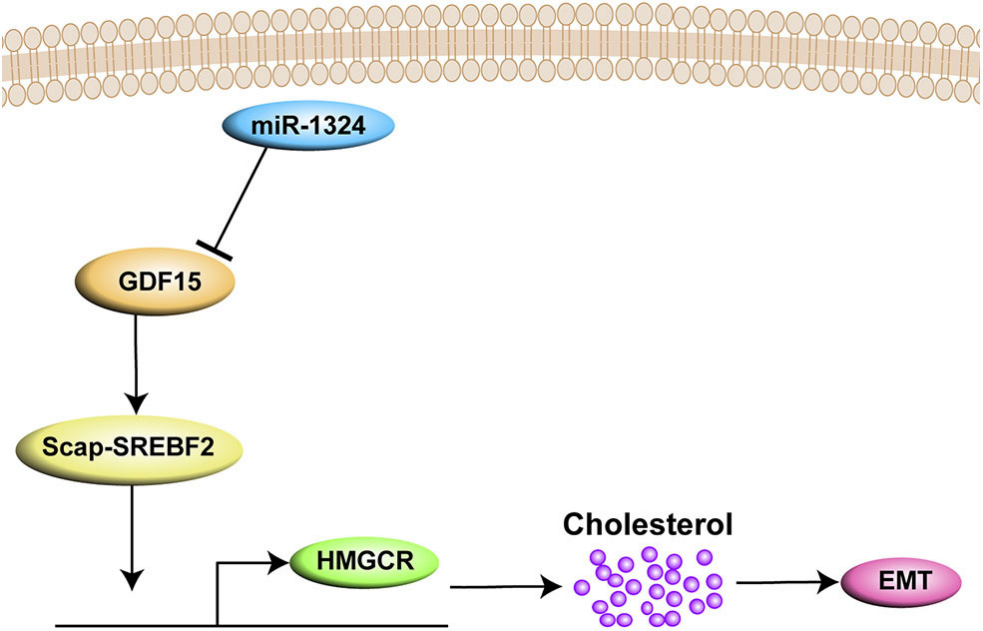
A graph illustrates that SCAP mediated GDF15-induced EMT of EC by regulating cholesterol metabolism. In addition, GDF15 was shown to be a direct target of miR-1324.

In summary, the present study demonstrates that SCAP mediates GDF15-induced invasion and metastasis of EC by regulating cholesterol metabolism. In addition, we found that GDF15 was a target of miR-1324 (Figure 7).

## Data Availability Statement

The raw data supporting the conclusions of this article will be made available by the authors, without undue reservation.

## Ethics Statement

The studies involving human participants were reviewed and approved by the Ethical Committee on Animal Experiments of Animal Care Committee of Zhongshan Hospital of Fudan University. The patients/participants provided their written informed consent to participate in this study. The animal study was reviewed and approved by the Ethical Committee on Animal Experiments of Animal Care Committee of Zhongshan Hospital of Fudan University.

## Author Contributions

GD, XH, SC, and CZ developed this study concept and designed. GD and XH analyzed *in vitro* experimental data and drafted this article. SJ, LN, and LM performed animal experiments and analyzed the data. SC and CZ supervised the research and provided critical review and revised version of this manuscript. All authors contributed to the article and approved the submitted version.

## Conflict of Interest

The authors declare that the research was conducted in the absence of any commercial or financial relationships that could be construed as a potential conflict of interest.
