# Supplementary figures and images for: SCAP Mediated GDF15-Induced Invasion and EMT of Esophageal Cancer
Source: Front Oncol. 2020 Oct 6;10:564785. doi: 10.3389/fonc.2020.564785 (PMC7573169; doi:10.3389/fonc.2020.564785)

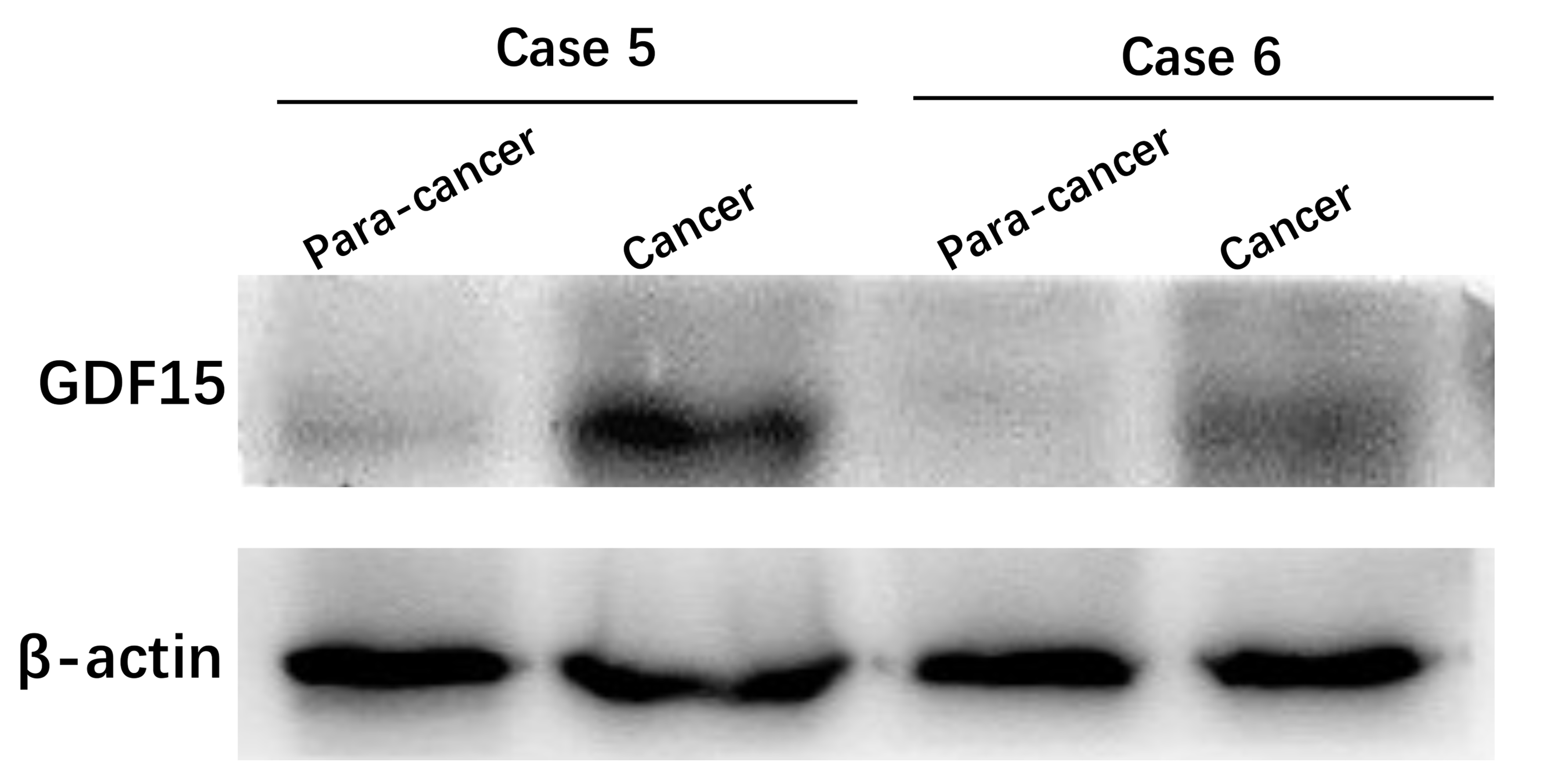

Supplement: Supplementary file 1 [file Image_1.TIF]

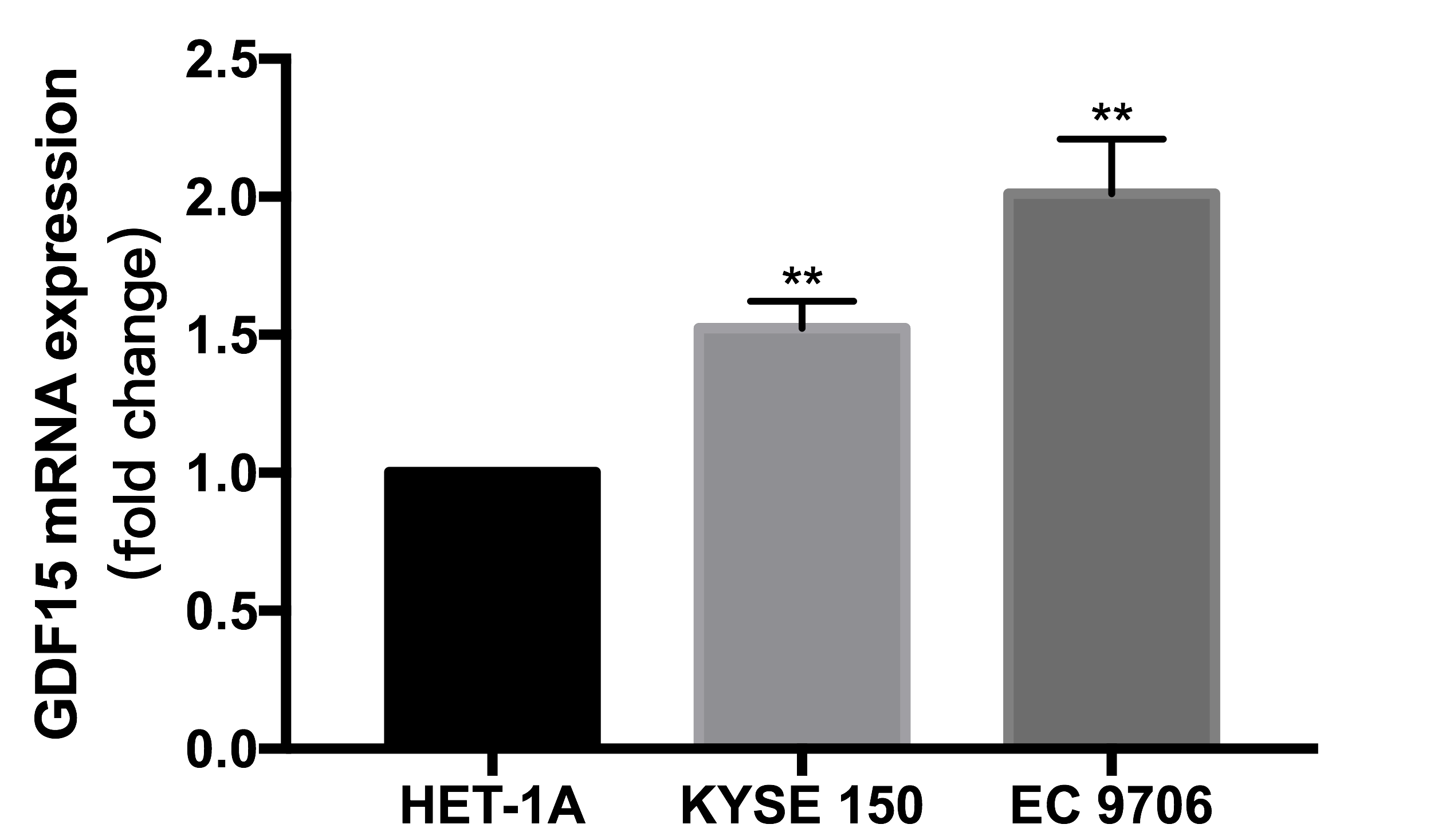

Supplement: Supplementary file 2 [file Image_2.TIFF]

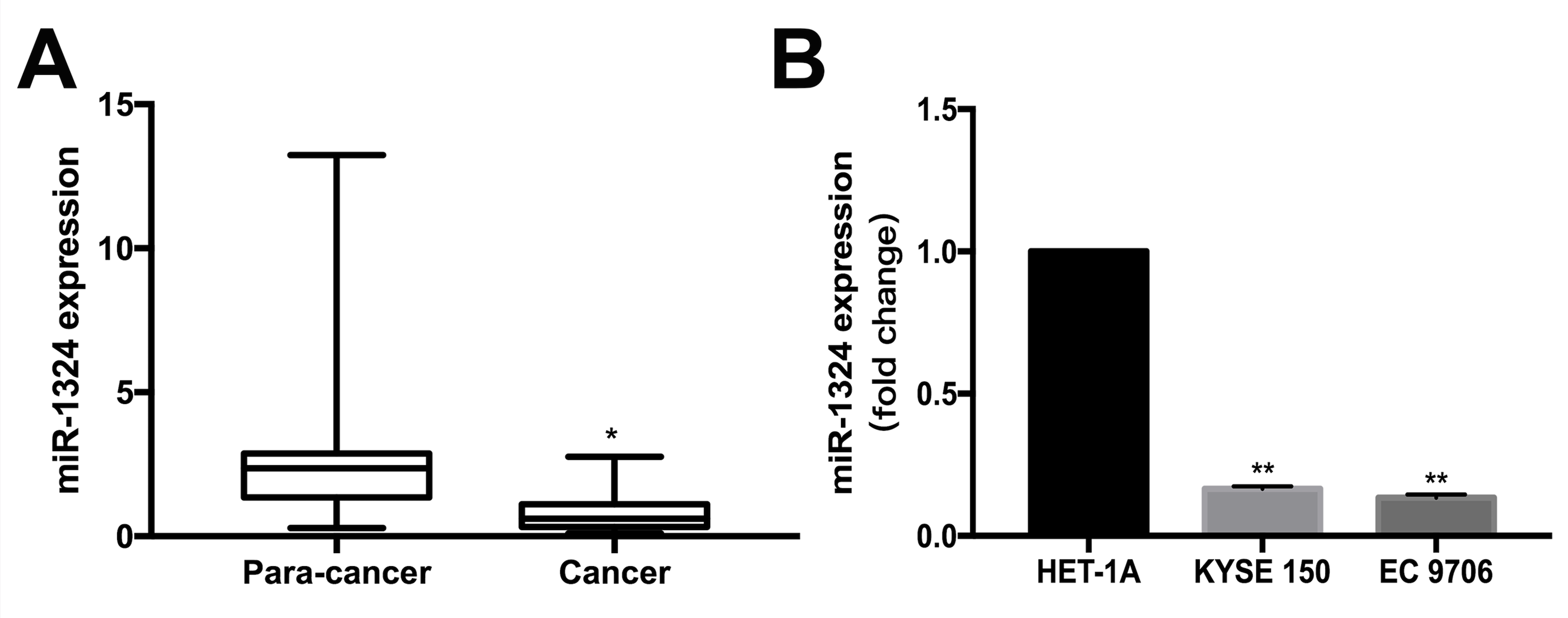

Supplement: Supplementary file 3 [file Image_3.TIF]
